# Positive and negative interspecific interactions between coexisting rice planthoppers neutralise the effects of elevated temperatures

**DOI:** 10.1111/1365-2435.13683

**Published:** 2020-10-04

**Authors:** Finbarr G. Horgan, Arriza Arida, Goli Ardestani, Maria Liberty P. Almazan

**Affiliations:** ^1^ EcoLaVerna Integral Restoration Ecology Kildinan Ireland; ^2^ Environment and Sustainable Resource Management University College Dublin Belfield, Dublin 4 Ireland; ^3^ International Rice Research Institute Metro Manila Philippines; ^4^ Department of Veterinary and Animal Sciences University of Massachusetts Amherst MA USA

**Keywords:** climate change, exploitation competition, facilitation, induced plant defences, interference competition, rice, volatiles

## Abstract

1. Global warming is often predicted to increase damage to plants through direct effects on insect herbivores. However, the indirect impacts of rising temperatures on herbivores, mediated through interactions with their biotic environment, could dampen these effects.
2. Using a series of reciprocal density experiments with gravid females and developing nymphs, we examined interspecific competition between two coexisting phloem feeders *Nilaparvata lugens* (BPH) and *Sogatella furcifera* (WBPH), on rice at 25 and 30°C.
3. WBPH performed better (i.e. adults survived longer, nymphs developed faster and grew larger) at 25°C and BPH (i.e. nymphs developed faster) at 30°C. However, contrary to predictions, WBPH had a greater effect in reducing oviposition and nymph performance in BPH at 30°C.
4. A decoupling of resource use by WBPH and its antagonistic effects on BPH at the higher temperature suggests that WBPH feeding induces host defences that reduce BPH fitness (i.e. interference competition). Meanwhile, BPH facilitated WBPH oviposition at 30°C and facilitated WBPH nymph performance at 25 and 30°C. Greater facilitation of feeding in WBPH nymphs by BPH at high densities suggests that mechanical damage and host responses to damage increased the fitness of the heterospecific nymphs.
5. Although BPH also facilitated egg‐laying by WBPH, intra‐ and interspecific crowding countered this facilitation at both temperatures. Simulated life tables for planthoppers at 25 and 30°C depicted significantly lower offspring numbers on rice infested by WBPH alone and from mixed BPH‐WBPH infestations than from infestations by BPH alone.
6. Our results indicate how interference competition—mediated through host plant defences—can increase ecosystem resilience to the warmer temperatures predicted under global climate change.

A free Plain Language Summary can be found within the Supporting Information of this article.

## Graphical abstract

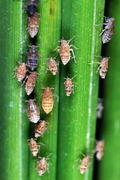

## 1 INTRODUCTION

Global temperatures have increased by 0.5–0.9°C over the last several decades. Based on global carbon dioxide (CO_2_) emissions, temperatures are predicted to increase a further 1.0–3.0°C before 2100 AD (Horgan, 2020; IPCC, 2014). Global warming directly affects ectothermic species in a number of ways: species may develop faster or grow larger during immature stages, they may increase their distribution ranges poleward or to higher altitudes or they may alter the seasons and routes of their migrations (Horgan, 2020; Hullé et al., 2010; Lu et al., 2012; Wu et al., 2019; Trnka et al., 2007). Compared to such direct effects, predicting the indirect impacts of temperature on herbivore populations as mediated through host plants, endosymbionts or natural enemies is a more challenging task—particularly for biodiverse ecosystems where large numbers of species interact across multiple trophic levels (Barton & Ives, 2014; Bidart‐Bouzat & Imeh‐Nathaniel, 2008; Horgan, 2020; Jeffs & Lewis, 2013; Meisner et al., 2014). Antagonistic interactions between species have the potential to dampen the positive direct effects of climate change on herbivores; for example, herbivore damage may actually decrease during periods of elevated temperature if the regulatory services provided by natural enemies increase to a greater extent than the (dis)services caused by herbivore populations (Hullé et al., 2010). In contrast, species interactions that facilitate herbivore survival and development have the potential to enhance the positive effects of climate change on herbivores (Barton & Ives, 2014). These contrasting outcomes are the result of condition‐dependent responses at the community level.

Phloem‐feeding insects are frequently used as models for climate change research (Han et al., 2019; Hullé et al., 2010; Meisner et al., 2014). Phloem feeders induce specific defence responses in the host, often with significant cross‐talk between response pathways that generates complex, plant‐mediated interactions between competing herbivores, or between the herbivores and the host plant's ephyphytes or pathogens (Denno et al., 2000; Matsumura & Suzuki, 2003; Satoh et al., 2009). Some detailed multi‐trophic studies have been conducted to elucidate the potential effects of elevated temperatures and other changes in climate on the population dynamics of phloem feeders and on their impacts on host plants, including crops (Barton & Ives, 2014; Han et al., 2019; Meisner et al., 2014). Such studies have shown that changes in climate can alter the outcomes of interactions between competing herbivores to affect herbivore community structure and the composition of plant communities (Lin et al., 2018; Ntiri et al. 2016; Schädler et al., 2007; Sun et al., 2009; Wang et al., 2018; Wang et al., 2020). Much of this previous research on the nature of temperature‐dependent interspecific interactions between herbivorous insects has focused primarily on interspecific competition for resources without assessing the effects of temperature on positive or negative interspecific interactions as mediated through plant defence pathways.

In the present study, we investigate temperature‐dependent positive (facilitation) and negative (interspecific competition) interactions between the brown planthopper (BPH), *Nilaparvata lugens*, and the whitebacked planthopper (WBPH) *Sogatella furcifera*, two phloem‐feeding planthoppers that co‐occur on rice, *Oryza sativa*, throughout Asia (Bottrell & Schoenly, 2012). Because WBPH is a superior competitor to BPH (Cheng et al., 2001; Srinivasan et al., 2016) and has lower optimal temperatures for nymph development and oviposition (Horgan, Arida, et al., 2020b), we predicted that the antagonistic effects of interspecific competition on BPH would be greater at a lower temperature (i.e. 25°C: close to optimum for WBPH) than at a higher temperature (i.e. 30°C: detrimental for WBPH, but not for BPH). Furthermore, because WBPH facilitation is associated with feeding activity by BPH (Cao et al., 2013; Matsumura & Suzuki, 2003; Srinivasan et al., 2016), we predicted that WBPH will perform better in the presence of BPH than when feeding alone, thereby achieving greater fitness (survival × reproduction) at higher temperatures than expected based on single species experiments. We used a series of reciprocal competition experiments to test these predictions. We assessed planthopper fitness in the presence of low (without intraspecific competition) and high (with intraspecific competition) densities of conspecifics, both with (+ interspecific competition) and without (− interspecific competition) heterospecifics at constant densities. Similar positive and negative interspecific effects under varying levels of competitor (WBPH) or facilitator (BPH) activity as related to the temperature optima for each species would suggest that feeding activities trigger plant‐mediated responses to impact heterospecifics, with resource depletion playing a relatively lesser role. We conducted the experiments using *indica* and *japonica* rice varieties based on observations that WBPH performs better on *japonica* varieties and BPH on *indica* varieties (Horgan, Srinivasan, et al., 2016). We discuss our results in light of the role of induced defences in determining herbivore and disease pressures on plants.

## 2 MATERIALS AND METHODS

### 2.1 Herbivore species

We used BPH and WBPH from colonies maintained at the International Rice Research Institute (IRRI) in the Philippines. The colonies were initiated in 2009 with >500 wild‐caught individuals of each species collected from Laguna Province (Philippines: 14°10′N, 121°13′E). Planthoppers of each species were reared continuously on the variety TN1 (≥30 days after sowing [DAS]) in three (WBPH) or five (BPH) wire mesh cages (91.5 × 56.5 × 56.5 cm; H × L × W). Individuals of the same species were frequently mixed between cages to avoid inbreeding. The colonies were kept under greenhouse conditions (26–37°C, 12:12 day:night) with feeding plants replaced every 3–5 days.

### 2.2 Plant materials

We used two rice varieties in our experiments. IR22 is an *indica* rice variety that is susceptible to BPH and WBPH populations from South and Southeast Asia. T65 is a *japonica* variety from Taiwan. The variety is highly susceptible to BPH and WBPH from South and Southeast Asia (Horgan et al., 2017). WBPH females often lay more eggs on T65 than on IR22 (Horgan, Srinivasan, et al., 2016). Both varieties are more susceptible than the natal variety TN1 to planthoppers from the Philippines (both BPH and WBPH; Ferrater et al., 2015; Horgan et al., 2017); although switching between natal and susceptible hosts can result in reduced planthopper fitness for several generations (Alam & Cohen, 1998; Ferrater et al., 2015). Seeds of the two varieties were acquired through the IRRI Germplasm Collection. For all experiments, the seed of T65 and IR22 were sown to #0 pots (5 × 2.5 cm: H × R) filled with paddy soil. The plants received no fertilisers and were not treated with any chemicals. At 15–18 DAS, the plants (one per pot) were placed under acetate cages (45 × 5 cm: H × R), each with a mesh top and side window. The cages were placed in the temperature chambers to allow the plants to acclimatise to the final experimental temperatures (i.e. either 25, 30 or 35°C). All planthopper infestations were to plants at 20 DAS. During the experiments, the plants were monitored for symptoms of hopper damage (i.e. yellowing or desiccation) to ensure that no plants died during the experiments. Initiating experiments with only a single 20‐day‐old seedling per pot intensified competition pressures without killing the plants.

### 2.3 Climate chamber experiments

Bioassays were conducted in environmental chambers with the Conviron CMP6050 Control System (Conviron). Temperature treatments were rotated between four separate chambers—changing the temperature settings after each experimental run. In some cases, one or two further runs were conducted by randomly assigning test temperatures to the chambers. Each replicate (i.e. run) included between one and five subsamples (i.e. rearing cages—see below) per variety, and per intra‐ and interspecific planthopper density, depending on the availability of plants and planthoppers and on available space in the climate chambers (i.e. up to a maximum of 200 cages per temperature per run: see Table S1). Subsamples were randomised within chambers. We conducted studies at 25 and 30°C based on apparent temperature optima for oviposition in WBPH and BPH respectively (Horgan, Arida, et al., 2020b). Initial experiments to investigate temperature effects on oviposition and nymph development were also conducted at 35°C. Relative humidity was maintained at 85% for all temperatures using an integrated fine‐mister.

#### 2.3.1 Assessment of temperature effects on planthopper oviposition

Oviposition by BPH and WBPH on IR22 and T65 was examined at three temperatures. Gravid females were collected from the BPH and WBPH colonies and carefully placed into the cages described above (one female per cage and one plant per cage) in environmental chambers set at 25, 30 or 35°C. Females were not acclimated, but were taken directly from the source colonies to the chambers during early morning. The females were allowed to feed and oviposit for up to 20 days. Plants were destructively sampled each day, noting the female condition (alive or dead) and transferring living females to fresh plants. Sampled plants were dissected under a stereo‐microscope to count egg clusters and eggs and the plants were then dried for >5 days in a forced draft over at 60°C and weighed. Egg production per female was calculated as the sum of eggs laid each day until the individual female had died (for further details see methods in Horgan, Arida, et al., 2020b). The experiment was replicated four times (i.e. 4 runs: Table S1).

#### 2.3.2 Assessment of temperature effects on nymph development

Neonate planthoppers were placed into cages (described above, each with one IR22 or T65 rice plant) at a density of 10 neonates per plant, using a handmade pooter. Neonates were not acclimated before the experiments. The neonates were collected from the source colonies within 6 hr of hatching and were transferred to the chambers during the early morning. The nymphs were allowed to feed and develop for 15 days in environmental chambers set at 25, 30 and 35°C. After 15 days, the plants were destructively sampled and the number of surviving nymphs and their developmental stages recorded. The nymphs and plants were then dried in a forced draft oven at 60°C for >5 days and weighed. The experiment was replicated five times (i.e. 5 runs: Table S1).

#### 2.3.3 Interspecific competition between ovipositing planthoppers

Plants and cages were prepared as described above. To calibrate the competition experiments we first assessed the intensity of intraspecific competition for oviposition at a range of densities and at the different temperatures. A high density, at which competition was observed (based on reduced egg‐laying per individual), but without killing the host plant, was selected for experiments at each temperature. Gravid female planthoppers were taken directly from source colonies and placed in cages, each with a single IR22 or T65 rice plant as follows: in a first series of cages, BPH densities were kept constant at four females (for experiments at 25°C) or six females (for experiments at 30°C) per plant, whereas densities of WBPH in the same cages were set at 0, 2 or 12. A set of cages with WBPH at 2 and 12 per plant, but without heterospecifics was also maintained (Table S1). In the second series of cages, WBPH densities were kept constant at four or six females per plant (for experiments at 25 and 30°C, respectively), whereas densities of BPH females were set at 0, 2 and 12 per plant. A set of cages with BPH females at 2 and 12 per plant, but without heterospecifics was also maintained (Table S1). Higher densities of planthoppers in the experiments at 30°C were required to counter increased plant growth rates at the higher temperature (unpubl. data) and thereby ensure that competition occurred.

The experiment was replicated five times (*N* = 5: Table S1) in chambers at 25 and 30°C. During each run, plants were all infested on the same day. Females were allowed to feed and oviposit for seven days after which, the plants were cut above the soil and were dissected under a stereo‐microscope (10× magnification) to count egg clusters and eggs. Eggs of BPH and WBPH can be distinguished based on their colour and form. Plants were dried in a forced draft oven at 60°C for >5 days before weighing.

#### 2.3.4 Interspecific competition between planthopper nymphs

Neonate planthoppers were taken directly from source colonies within 6 hr of hatching and placed in cages, each with a single IR22 or T65 rice plant. Based on preliminary bioassays, a density of 10–25 nymphs per plant was required to ensure intraspecific competition occurred between nymphs, but without killing the host plant. In a first series of cages, BPH densities were kept constant at 10 per plant, whereas densities of WBPH in the same cages were set at 0, 5 and 15 per plant. A set of cages with WBPH at 5 and 15 per plant, but without heterospecifics was also maintained (Table S1). In the second series of cages, WBPH densities were kept constant at 10 per plant, whereas densities of BPH were set at 0, 5 and 15 per plant. A set of cages with BPH at 5 and 15 per plant, but without heterospecifics was also maintained (Table S1). The highest density of planthoppers (both species) on plants in any treatment was 25 individuals.

The infested plants were maintained in climate chambers set at 25°C or 30°C. The experiment was replicated five times (*N* = 5). During each run, plants were all infested on the same day. Nymphs were allowed to feed and develop for 15 days after which, the number of survivors was recorded. The survivors and plants, cut above the soil, were then collected and dried at 60°C for >5 days in a forced draft oven before being weighed.

### 2.4 Data analyses

Adult survival, oviposition and nymph performance (survival, development and biomass) during initial experiments were analysed using univariate general linear models (LMs) with temperature, variety and their interactions as main factors. Plant weight at the end of the experiments was initially included as a covariate in the analyses, but was removed because it had no significant effect. Life‐history parameters were also compared between species by univariate LM (Table S2). Interspecific effects on total eggs laid and nymph performance were analysed separately for planthopper species using univariate LM with conspecific density, presence/absence of the heterospecific, temperature, variety and their interactions as main factors. Egg numbers and nymph biomass data were log‐transformed, nymph survival was logit‐transformed (Warton & Hui, 2011), and development was ranked before analyses. Residuals were plotted and found to be normal and homogeneous after parametric analyses. Interspecific effects were assessed for both species in each set of experiments, that is, with one species as the main test species (with varying densities) and the second as the heterospecific competitor (with constant densities). We present results only for the main species in the main text; corresponding results for heterospecific competitors are presented in Figure S1 and Tables S3 and S4). To further assess the relative impacts of BPH or WBPH on heterospecific egg laying and nymph weights, we also calculated an ‘index of competition effect’ (see Supporting Information).

The potential effects of interspecific competition on planthopper populations at 25 and 30°C were assessed using simulations based on constructed life tables according to Liu and Han (2006). We assumed an initial cohort of 200 neonates in each population and determined the numbers of offspring entering the next generation based on measured survival and reproduction parameters. Offspring numbers were calculated as the product of the initial population size in each generation and the proportion of nymphs surviving, the proportion that were female, the number of eggs laid per female, and the proportions of eggs that hatch. Initial cohorts were either 200 BPH alone, 200 WBPH alone, or BPH and WBPH mixed (100 of each species). Values for nymph survival were based on experiments with intraspecific competition only (density = 15), or interspecific competition (density = 15 with heterospecifics present at 10 per plant; *N* = 5). The sex‐ratios of survivors at each temperature were based on experiments reported by Horgan, Arida, et al. (2020b). Previous experiments with BPH reported by Horgan, Naik, et al. (2016) suggest that sex‐ratios are not significantly affected by competition. Values for female fecundity were based on experiments with intraspecific competition only (density = 12), or interspecific competition (density = 12 with heterospecifics present at between 4 and 6 per plant; *N* = 5). We applied values of 0.900 and 0.930 for BPH hatchability at 25 and 30°C, respectively, based on information in Horgan, Arida, et al. (2020b). We used WBPH hatchability values of 0.961 and 0.972 for 25 and 30°C, respectively, based on data presented by Tu et al. (2013) for planthopper eggs at 25 and 28°C. We simulated populations for three generations (i.e. the typical number of generations during a single crop Cheng et al., 2001). Offspring numbers were analysed using a repeated measures LM with cohort‐type (BPH, WBPH, or mixed) as the main factor. The analysis assumes that the presence of heterospecifics had a greater impact than the density of heterospecifics in the experiments (see below).

## 3 RESULTS

### 3.1 Initial oviposition experiments

**FIGURE 1:**
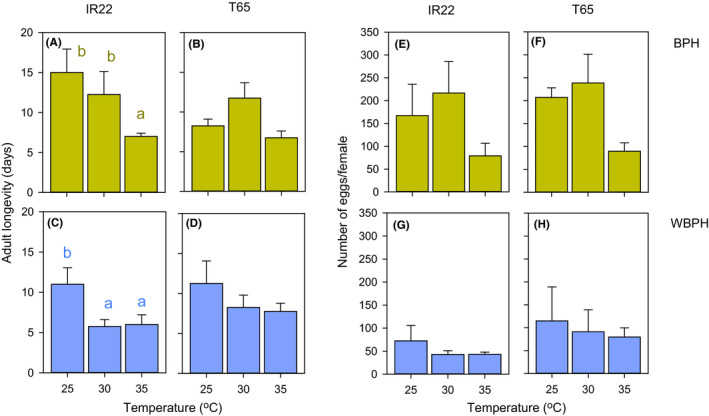
Longevity of adult female BPH (A, B) and WBPH (C, D) on IR22 (A, C) and T65 (B, D) in environmental chambers at three constant temperatures. The corresponding numbers of eggs laid by BPH (E, F) and WBPH (G, H) on IR22 (E, G) and T65 (F, H) are also presented. Error bars are indicated (*N* = 4). Numbers are based on survival and oviposition by females for up to 20 days at each temperature. Lowercase letters indicate homogenous temperature groups (Tukey: *p* > 0.05)

BPH females laid more eggs than WBPH females during the experiments (Figure 1; Table S2). BPH survived longer at 25 and 30°C than at 35°C, whereas WBPH females survived longer at 25°C than at the other temperatures (Figure 1A–D; Table 1). BPH survived longer on IR22 plants, but variety had no effect on WBPH survival (Figure 1A–D; Table 1). Egg laying by either planthopper species was not affected by temperature (Table 1). WBPH laid more eggs on T65 plants (Figure 1G,H); but there was no effect of variety on egg laying in BPH (Figure 1E,F). However, there was a significant [temperature*variety] interaction for BPH because the numbers of eggs laid at 35°C were not different from at 25°C on IR22, but they were significantly lower than at 25°C on T65 (Figure 1; Table 1).

**TABLE 1:** Results of general linear models for planthopper oviposition and nymph performance on two rice varieties in environmental chambers at three constant temperatures (see Figures 1 and 2; Table S2)

Sources of variation	*df* ^a^	*F*‐values^b^					
		Adult longevity	Eggs laid	Nymph survival	Development to fifth instar	Total planthopper weight	Average planthopper weight
BPH							
Temperature (*T*)	2	8.467***	1.994	18.329***	47.797***	260.452***	108.707***
Variety (*V*)	1	4.863*	4.751*	0.001	0.001	0.137	0.246
Replicate	3 [4]	6.542***	4.265*	2.678	8.051***	2.316	3.292*
T*V	2	3.518	8.461***	0.615	0.818	0.319	0.437
Error	15 [20]						
WBPH							
Temperature (*T*)	2	4.786*	0.989	11.415***	48.198***	410.048***	253.659***
Variety (*V*)	1	1.381	5.194*	1.103	0.281	0.136	0.119
Replicate	3 [4]	2.261	5.260**	2.003	2.253	5.010**	4.960**
T*V	2	0.268	0.053	1.418	0.281	3.181	1.584
Error	15 [20]						

^a^ *df* in square parentheses are for nymph survival experiments (i.e. nymph survival, development to fifth instar, total planthopper weight and average planthopper weight).

^b^ ***p ≤ 0.001, **p ≤ 0.01, *p ≤ 0.05; data for survival were logit‐transformed, data for development were ranked and egg numbers and weights were log‐transformed before analysis.

### 3.2 Initial nymph experiments

**FIGURE 2:**
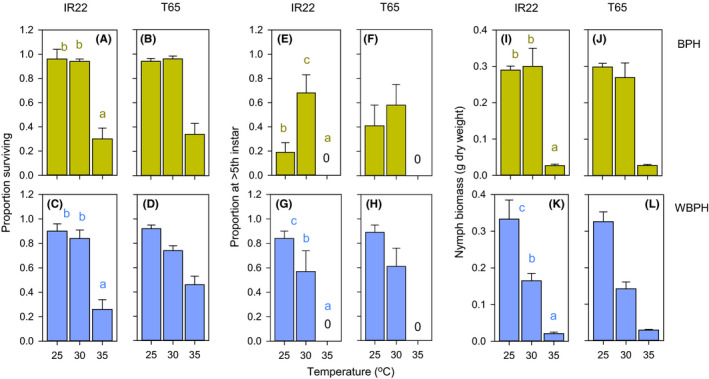
Survival of BPH (A, B) and WBPH (C, D) nymphs at three temperatures on IR22 (A, C) and T65 (B, D), with development of BPH (E, F) and WBPH (G, H) and dry weight per individual BPH (I, J) and WBPH (K, L) on IR22 (E, G, I, K) and T65 (F, H, J, L). Standard errors are indicated (*N* = 5). Lowercase letters indicate homogenous temperature groups (Tukey: *p* > 0.05)

Nymphs of both species had low survival and weight gain when reared at 35°C (Figure 2A–D; Table 1). BPH nymphs developed faster at 30°C (Figure 2E,F; Table 1); meanwhile, WBPH nymphs developed faster and grew larger at 25°C than at the higher temperatures (Figure 2G–L; Table 1). Nymph development was generally faster in WBPH, compared to BPH (Figure 2E–H), but nymphs were smaller (Figure 2I–L; Table S2).

### 3.3 Interspecific competition during oviposition

**FIGURE 3:**
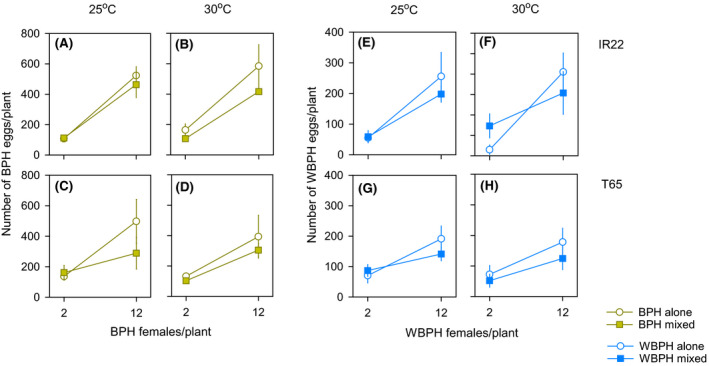
Oviposition during reciprocal interspecific competition experiments conducted at 25°C (A, C, E, G) and 30°C (B, D, F, H). In one set of experiments, BPH female densities were increased in the presence of constant densities (four per plant or six per plant at 25 and 30°C, respectively) of WBPH females (A–D). In the second set, WBPH female densities were increased in the presence of constant densities (four and six females per plant at 25 and 30°C, respectively) of BPH females (E–H). Results indicate the total numbers of eggs per planthopper for each species. Experiments were conducted using the varieties IR22 (A, B, E, F) and T65 (C, D, G, H). Brown symbols and lines indicate BPH and blue symbols and lines indicate WBPH. Standard errors are indicated (*N* = 5). The numbers of eggs laid by planthoppers at constant densities in the same experiments are presented in Figure S1 and results as total eggs per plant are presented in Figure S2

The total numbers of eggs laid by females of each species per plant are presented in Figure S2 and the numbers of eggs per individual are presented in Figure 3. Fewer eggs were laid per BPH female at high densities (*F*_1,59_ = 39.858, *p* < 0.001) and in the presence of WBPH (*F*_1,59_ = 4.539, *p* = 0.037; Figure 3A–D). There was no effect of temperature on BPH egg numbers in the presence or absence of WBPH (*F*_1,59_ = 0.243, *p* = 0.624; Figure 3A–D).

Fewer eggs were produced per WBPH female at 30°C (*F*_1,59_ = 8.368, *p* < 0.001; Figure 3E–H; see also Figure S1). Fewer eggs were produced per WBPH female at a density of 12 per plant (*F*_1,59_ = 12.788, *p* < 0.001), but this was not affected by the presence of the heterospecific competitor (*F*_1,59_ = 0.452, *p* = 0.504; Figure 3E–H). There were several significant interactions: the presence of heterospecifics had no effect on egg numbers at low WBPH densities, but caused a reduction in egg numbers at high densities (*F*_1,59_ = 8.876, *p* = 0.004); the presence of heterospecifics had a greater effect in facilitating WBPH oviposition on IR22 than on T65, producing a significant [variety*heterospecific presence] interaction (*F*_1,59_ = 6.886, *p* = 0.011); and WBPH egg numbers were higher on IR22 at a density of two females per plant, and at 25°C producing a significant [variety*temperature*WBPH density] interaction (*F*_1,59_ = 7.221, *p* = 0.009). Greater facilitation of WBPH egg laying by BPH at the low WBPH density, on IR22 and at 30°C (Figure 3F) produced a significant four factor interaction (*F*_1,59_ = 8.024, *p* = 0.006). The presence of heterospecifics had a greater effect in reducing the number of eggs laid by BPH than by WBPH (see Figure S3; Table S5).

### 3.4 Interspecific competition between planthopper nymphs

**FIGURE 4:**
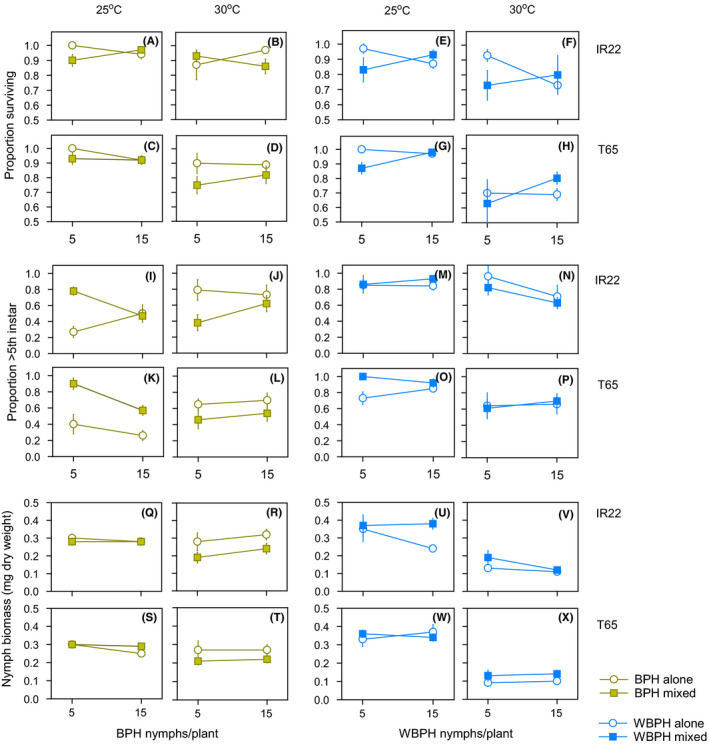
Nymph survival during reciprocal interspecific competition experiments conducted at 25°C (A, C, E, G) and 30°C (B, D, F, H) with corresponding development of nymphs at 25°C (I, K, M, O) and 30°C (J, L, N, P), and average nymph dry weights at 25°C (Q, S, U, W) and 30°C (R, T, V, X). The experiments were conducted on varieties IR22 (A, B, E, F, I, J, M, N, Q, R, U, V) and T65 (C, D, G, H, K, L, O, P, S, T, W, X). Brown symbols and lines indicate BPH and blue symbols and lines indicate WBPH. Standard errors are indicated (*N* = 5). Survival, development and weight gain for nymphs at constant densities in the same experiments are presented in Figure S1 and results as total nymph biomass per plant are presented in Figure S5

BPH nymph survival was not affected by temperature or the presence/absence of heterospecifics (Figure 4A–D; Table S4). WBPH nymph survival declined at the higher temperature (*F*_1,74_ = 22.033, *p* < 0.001) and declined in the presence of BPH (*F*_1,74_ = 4.117, *p* = 0.046). However, the effect was greater at a density of 5 WBPH per plant than at 15 per plant (*F*_1,74_ = 7.736, *p* = 0.007; Figure 4E–H). Development of BPH nymphs was faster in the presence of WBPH nymphs, but only at 25°C (*F*_1,74_ = 47.909, *p* = 0.037), the effect was greatest under conditions of low conspecific densities, producing a significant [temperature*conspecific density*heterospecific presence] interaction (*F*_1,74_ = 10.378, *p* = 0.002; Figure 4I–L). WBPH development was faster at 25°C (*F*_1,74_ = 12.519, *p* < 0.001) and faster at that temperature in the presence of BPH nymphs (*F*_1,74_ = 4.718, *p* = 0.033; Figure 4M–P).

BPH nymphs attained higher weights at 30°C (*F*_1,74_ = 9.880, *p* = 0.002), and in the absence of heterospecifics (*F*_1,74_ = 5.318, *p* = 0.024; Figure 4Q–T). The presence of WBPH had a greater effect in reducing BPH nymph biomass at 30°C than at 25°C as indicated by a significant [temperature*heterospecific presence] interaction (*F*_1,74_ = 10.853, *p* < 0.001; Figure 4Q–T). A significant [temperature*conspecific density] interaction (*F*_1,74_ = 6.097, *p* = 0.016) was due to a slight divergence in nymph weights at higher densities, at 30°C (Figure 4R,T). Totals for nymph biomass per plant are indicated in Figure S4.

WBPH nymph weights were lower at 30°C (*F*_1,74_ = 227.640, *p* < 0.001); but increased in the presence of heterospecifics (*F*_1,74_ = 8.337, *p* = 0.005; Figure 4U–X). Strong facilitation of WBPH nymph weight gain by heterospecifics under conditions of intraspecific crowding, on IR22, at 25°C produced a significant [variety*temperature*hereteospecific*conspecific density] interaction (*F*_1,74_ = 5.291, *p* = 0.024; Figure 4U–X). Losses in nymph biomass due to interspecific competition were greater for BPH (*F*_1,80_ = 8.475, *p* = 0.005) and greatest at 30°C (*F*_1,80_ = 5.344, *p* = 0.023; Figure S5; Table S5).

### 3.5 Life tables

**FIGURE 5:**
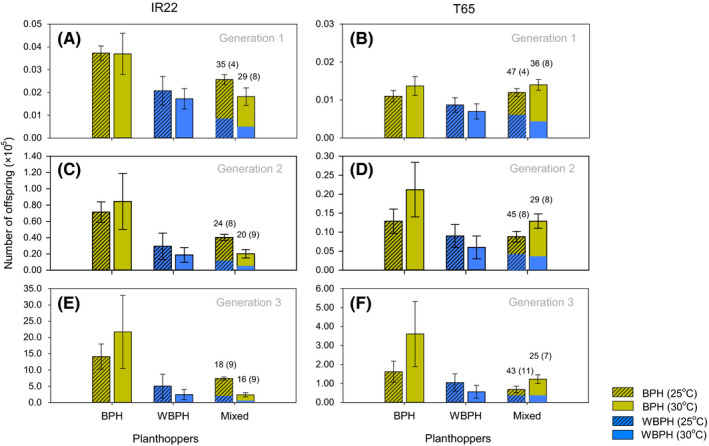
Estimated number of offspring from 200 BPH nymphs, 200 WBPH nymphs or from a mix of nymphs of both species (100 + 100) after three generations (first generation = A, B, second = C, D, third = E, F) on IR22 (A, C, E) and T65 (B, D, F) at 25°C (shaded) or 30°C (solid). Numbers associated with mixed cohorts indicate the percentage of offspring that were WBPH. Estimates are based on constructed life tables adapted from Liu and Han (2006) using survival rates and fecundities from the competition experiments. Standard errors are indicated by error bars (numbers of hoppers) and in parentheses (percentage of WBPH in the mixed cohorts; *N* = 5)

Simulated offspring numbers increased over three generations (*F*_2,96_ = 1,260.730, *p* < 0.001), however, there was a significant [generation*cohort type] interaction (*F*_4,96_ = 9.146, *p* < 0.001) because of similar numbers of offspring during the first generation, but lower numbers in WBPH (alone) cohorts or mixed BPH/WBPH cohorts compared to cohorts with BPH (alone) during the second and third generations (Figure 5). There was also a significant [generation*variety] interaction (*F*_2,96_ = 8.799, *p* < 0.001) because of similar numbers of offspring on both varieties during the first generation, but lower numbers on T65 in subsequent generations. Fewer offspring were produced on T65 than on IR22 (between subject effect: *F*_1,48_ = 8.026, *p* = 0.007), largely due to lower oviposition by BPH on T65 during the environmental chamber experiments (Figure 5). There was no significant temperature effect, or significant interactions between temperature and between‐subject or within‐subject factors (all *F* ≤ 0.816). Fewer offspring from WBPH and mixed cohorts than from BPH cohorts (between subject effect: *F*_2,48_ = 9.850, *p* < 0.001) suggests that the presence of WBPH on plants would reduce total offspring numbers irrespective of temperature and despite lower performance by the species at 30°C.

## 4 DISCUSSION

Horgan, Arida, et al. (2020b) have recently reviewed information on the temperature profiles of BPH and WBPH. These authors indicated that, whereas the two species have similar temperature tolerances and similar thresholds for development, WBPH performs better (i.e. higher fecundity, faster development, greater weight gain) at lower temperatures and BPH at higher temperatures. Furthermore, temperatures of ≈35°C were detrimental for the eggs and nymphs of both species (see also Park et al., 2013; Piyaphongkul et al., 2012). Our results for adult survival, oviposition and nymph growth and development largely followed these trends. Based on observed differences in planthopper responses to temperatures and because BPH facilitates WBPH feeding and development (Cao et al., 2013; Matsumura & Suzuki, 2003; Srinivasan et al., 2016), we predicted that the presence of BPH would counter the detrimental effects of high temperatures (i.e. 30°C) on WBPH. Furthermore, because it is a superior competitor to BPH (Cheng et al., 2001; Srinivasan et al., 2016), we predicted that WBPH would have a greater impact on BPH populations at 25°C, which is close to optimal for WBPH, than at 30°C. However, based on our initial experiments, temperature effects on oviposition were less pronounced than the effects on nymph growth and development. This was reflected in the relative strength of responses by planthopper adults and nymphs of both species to crowding under different temperatures in our experiments.

### 4.1 Effects of temperature on the facilitation of WBPH by BPH

Facilitation of WBPH oviposition by BPH was greatest at low planthopper densities (both conspecific and heterospecific), particularly on IR22, and at 30°C (i.e. on the lower quality host and at the less favourable temperature for WBPH: Figure 3F; Figure S1G,H). This supported our prediction that interspecific facilitation would assist WBPH in overcoming the direct, negative effects of higher temperatures. It also promoted WBPH oviposition on a less favoured host. However, an apparent lack of facilitation at higher female densities suggests that intra‐ and interspecific competition countered the effects of facilitation on oviposition, particularly at the higher temperature (Figure S1G,H). Because planthoppers oviposit within relatively localised areas near the base of the rice stem (Cheng et al., 2001) and because BPH females will oviposit during darkness, whereas WBPH females do not (Horgan, et al., 2016), we suggest that this was mainly due to competition for stem space.

**FIGURE 6:**
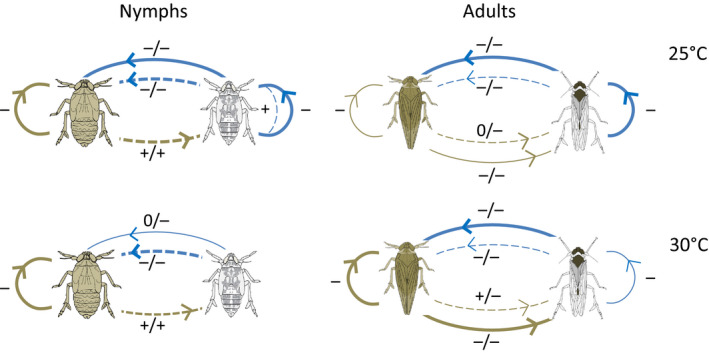
Exploitation competition (solid lines) and plant‐mediated interference competition or facilitation (dashed lines with ‘−’ or ‘+’, respectively) between BPH (brown planthopper) and WBPH (white planthopper) at 25°C and 30°C as indicated by changes in nymph biomass or eggs per female. Exploitation competition is identified by greater negative impacts at high conspecific (intraspecific competition) or heterospecific (interspecific competition) densities. Interference competition and facilitation are identified by negative and positive effects, respectively, on heterospecifics, regardless of planthopper densities. −/+ indicates a negative effect at low heterospecific density, but positive effect at high heterospecific density, etc.; ‘0’ signifies no effect (see also Figure S6)

Facilitation by BPH also improved WBPH nymph performance at 30°C. However, in contrast to the facilitation of WBPH oviposition, the facilitation of nymphs was most apparent where BPH densities were high (Figure 4E–H; Figure S1W,X). Facilitation of WBPH feeding is associated with BPH‐induced physical and biochemical changes to the rice plant. For example, based on EPG recordings, Cao et al. (2013) have shown that WBPH locate phloem more quickly and tend to suck phloem for longer on plants previously infested by BPH. Furthermore, BPH feeding induces the production of (*Z*)‐3‐hexenal in rice (Wang et al., 2008); the volatile (*Z*)‐3‐hexanal is associated with susceptibility to WBPH (Wang et al., 2015). Because these factors depend on the magnitude and nature of BPH damage to rice, they are decoupled from the direct effects of temperature on WBPH (Figure 6) and may be expected to vary depending on the conditions (e.g. resistance, age, etc.) of the host plant, or on the feeding history of the facilitator (i.e. whether BPH had developed on a resistant or susceptible natal host: Ferrater & Horgan, 2016; Horgan, et al., 2016; Pan et al., 2016).

### 4.2 Effects of temperature on interspecific competition

Based on differences in the performance of BPH and WBPH at 25 and 30°C, we expected WBPH to have a lower impact on BPH at the higher temperature. However, contrary to predictions, WBPH had its greatest negative impact on BPH oviposition, nymph survival and nymph weight gain at 30°C (Figure 6). Egg laying in both species was reduced by intra‐ and interspecific crowding. However, the negative effects of competition with heterospecifics for oviposition space were consistently greater on BPH than on WBPH (as indicated by the index of competition) and were greater at 30°C, despite WBPH laying fewer eggs at that temperature (Figure S1G,H). We suggest that intense intraspecific competition between ovipositing BPH at the higher temperature, together with the added impact of interspecific crowding, resulted in the relatively larger decline in BPH egg numbers per female at 30°C compared to 25°C (Figure 6).

The rate of development of BPH nymphs increased in the presence of WBPH at 25°C, but not at 30°C where the opposite tended to occur (Figure 4I–L; Figure 6). Our results suggest that BPH development accelerated under the unfavourable conditions created by intense competition for resources with WBPH at the lower temperature (e.g. see biomass of WBPH nymphs at 25 and 30°C in the same cages as presented in Figure S1W,X). BPH nymph survival and biomass declined in the presence of WBPH, but the effect was again greatest at 30°C (Figure 4A–D,Q–T), despite the deleterious effects of the high temperature on WBPH nymphs in the same cages (Figure S1K,L,O,P,W,X). This decoupling of resource use by WBPH from its antagonistic impact on the heterospecific indicates that the effects were due to interference competition mediated through the host plant. A consistently greater impact of WBPH on BPH nymph performance at 30°C compared to 25°C, suggests that intraspecific competition between BPH nymphs or increased activity, including increased phloem probing, further enhanced the negative effects of interspecific competition on BPH (Figure 6; Figure S5). Plant defences may also have functioned more effectively under the higher temperature.

### 4.3 Potential mechanisms of interference competition

WBPH has previously been shown to induce rice defences against *Magnaporthe grisea* (Kanno & Fujita, 2003; Kanno et al., 2005) and *Xanthomonas oryza* pv. *oryzae* (Gomi et al., 2010; Satoh et al., 2009), the causal agents of rice blast and bacterial blight respectively. WBPH herbivory stimulates the production of salicyclic Acid (SA) and jasmonic Acid (JA) involved in the rice plant's general defence system (Kanno et al., 2012). Attacks also stimulate the production and release of green leaf volatiles, including the (*Z*)‐3‐hexenal that increases rice susceptibility to the planthopper (Gomi et al., 2010; Wang et al., 2015), and induces the accumulation of defensive rice flavonoids and diterpenoids (Kanno et al., 2012). This induction of defences that is known to function against plant diseases probably also plays a role in the reduction of BPH fitness by WBPH on the same plants, particularly since the effects can be seen in plants after prior WBPH infestation. For example, in a study by Matsumura and Suzuki (2003), prior feeding by WBPH delayed the development of BPH nymphs by about 1 day (ca 7%). The greater impact of WBPH on BPH nymphs at elevated temperatures (i.e. 30°C compared to 25°C) may be related to a greater induction of such host defences at high temperatures. For example, in a review by Bidart‐Bouzat and Imeh‐Nathaniel (2008) warmer temperatures were associated with increased production of volatile organic compounds, similar to those that facilitate WBPH and are antagonistic to plant pathogens. Higher feeding rates at elevated temperatures can overwhelm plant defences (Havko et al., 2020; Horgan, Arida, et al., 2020a), but they could also lead to greater plant resistance at higher temperatures where planthoppers ingest larger quantities of plant defensive chemicals.

### 4.4 Concluding remarks

In our mixed‐species experiments, higher temperatures had a net beneficial effect on rice by reducing the fitness of a superior competitor (WBPH), but maintaining the deleterious effects of that competitor on a high temperature‐tolerant heterospecific. Our simulated life tables depicted a significantly slower build‐up of planthoppers on rice infested with WBPH alone or with mixed BPH‐WBPH cohorts, compared to rice infested with BPH alone (Figure 5). Because BPH is favoured by the higher temperatures that are now more prevalent in some rice‐growing regions (Horgan, Arida, et al., 2020a, 2020b), then maintaining WBPH as an antagonist may ultimately be more beneficial than controlling it (e.g. Satoh et al., 2009), particularly since BPH is a more damaging herbivore (Horgan, Arida, et al., 2020b). The outcome of the combined positive and negative interactions between BPH and WBPH indicates one mechanism by which the maintenance of biodiversity promotes stability in herbivore assemblages and thereby increases the resilience of ecosystems to the warmer temperatures predicted under global climate change.

## AUTHORS' CONTRIBUTIONS

F.G.H. conceived the manuscript and designed the methodology; A.A., G.A. and M.L.P.A. maintained plant and insect materials and conducted the experiments; F.G.H., A.A., G.A. and M.L.P.A. compiled the data; F.G.H. analysed the data and prepared figures; F.G.H. led the writing of the manuscript. All authors edited and reviewed the manuscript before submission.

## Supporting information

Supplementary Material

Supplementary Material

## Data Availability

Data are deposited in the Dryad Digital Repository https://doi.org/10.5061/dryad.m37pvmd0h (Horgan, Arida, et al., 2020c).

## References

[fec13683-bib-0001] Alam, S. N. , & Cohen, M. B. (1998). Durability of brown planthopper, *Nilaparvata lugens*, resistance in rice variety IR64 in greenhouse selection studies. Entomologia Experimentalis et Applicata, 89, 71–78. 10.1046/j.1570-7458.1998.00383.x

[fec13683-bib-0002] Barton, B. T. , & Ives, A. R. (2014). Direct and indirect effects of warming on aphids, their predators, and ant mutualists. Ecology, 95, 1479–1484. 10.1890/13-1977.1 25039213

[fec13683-bib-0003] Bidart‐Bouzat, M. G. , & Imeh‐Nathaniel, A. (2008). Global change effects on plant chemical defenses against insect herbivores. Journal of Integrative Plant Biology, 50, 1339–1354. 10.1111/j.1744-7909.2008.00751.x 19017122

[fec13683-bib-0004] Bottrell, D. G. , & Schoenly, K. G. (2012). Resurrecting the ghost of green revolutions past: The brown planthopper as a recurring threat to high‐yielding rice production in tropical Asia. Journal of Asia‐Pacific Entomology, 15, 122–140. 10.1016/j.aspen.2011.09.004

[fec13683-bib-0005] Cao, T.‐T. , Lü, J. , Lou, Y.‐G. , & Cheng, J.‐A. (2013). Feeding‐induced interactions between two rice planthoppers, *Nilaparvata lugens* and *Sogatella furcifera* (Hemiptera: Delphacidae): Effects on feeding and honeydew excretion. Environmental Entomology, 42, 1281–1291. 24468558 10.1603/EN12171

[fec13683-bib-0006] Cheng, J. , Zhao, W. , Lou, Y. , & Zhu, Z. (2001). Intra‐and inter‐specific effects of the brown planthopper and white backed planthopper on their population performance. Journal of Asia‐Pacific Entomology, 4, 85–92. 10.1016/S1226-8615(08)60108-9

[fec13683-bib-0007] Denno, R. F. , Peterson, M. A. , Gratton, C. , Cheng, J. , Langellotto, G. A. , Huberty, A. F. , Finke, D. L. (2000). Feeding‐induced changes in plant quality mediate interspecific competition between sap‐feeding herbivores. Ecology, 81, 1814–1827. 10.1890/0012-9658(2000)081[1814:FICIPQ]2.0.CO;2

[fec13683-bib-0008] Ferrater, J. B. , & Horgan, F. G. (2016). Does *Nilaparvata lugens* gain tolerance to rice resistance genes through conspecifics at shared feeding sites? Entomologia Experimentalis et Applicata, 160, 77–82. 10.1111/eea.12454

[fec13683-bib-0009] Ferrater, J. B. , Naredo, A. I. , Almazan, M. L. P. , de Jong, P. W. , Dicke, M. , & Horgan, F. G. (2015). Varied responses by yeast‐like symbionts during virulence adaptation in a monophagous phloem‐feeding insect. Arthropod‐Plant Interactions, 9, 215–224. 10.1007/s11829-015-9373-0

[fec13683-bib-0010] Gomi, K. , Satoh, M. , Ozawa, R. , Shinonaga, Y. , Sanada, S. , Sasaki, K. , Matsumura, M. , Ohashi, Y. , Kanno, H. , Akimitsu, K. , & Takabayashi, J. (2010). Role of hydroperoxide lyase in white‐backed planthopper (*Sogatella furcifera* Horváth)‐induced resistance to bacterial blight in rice, *Oryza sativa* L. The Plant Journal, 61, 46–57. 19891707 10.1111/j.1365-313X.2009.04031.x

[fec13683-bib-0011] Han, Z. , Tan, X. , Wang, Y. , Xu, Q. , Zhang, Y. , Harwood, J. D. , & Chen, J. , (2019). Effects of simulated climate warming on the population dynamics of *Sitobion avenae* (Fabricius) and its parasitoids in wheat fields. Pest Management Science, 75, 3252–3259. 30993856 10.1002/ps.5447

[fec13683-bib-0013] Havko, N. E. , Kapali, G. , Das, M. R. , & Howe, G. A. (2020). Stimulation of insect herbivory by elevated temperature outweighs protection by the jasmonate pathway. Plants, 9, e172 10.3390/plants9020172 32024094 PMC7076421

[fec13683-bib-0014] Horgan, F. G. (2020). Potential for an impact of climate change on insect herbivory in cereal crops In K. Jabran , S. Florentine , & B. S. Chauhan (Eds.), Crop protection under climate change (pp. 101–144). Springer Nature.

[fec13683-bib-0015] Horgan, F. G. , Arida, A. , Ardestansi, G. , & Almazan, M. L. P. (2020a). Elevated temperatures dampen the effects of a highly resistant rice variety on the brown planthopper. Scientific Reports (accepted). 10.1038/s41598-020-80704-4 PMC7794346 33420350

[fec13683-bib-0016] Horgan, F. G. , Arida, A. , Ardestansi, G. , & Almazan, M. L. P. (2020b). Temperature‐dependent oviposition and nymph performance reveal distinct thermal niches of coexisting planthoppers with similar thresholds for development. PLoS One, 15, e0235506 10.1371/journal.pone.0235506 32603337 PMC7326231

[fec13683-bib-0017] Horgan, F. G. , Arida, A. , Ardestansi, G. , & Almazan, M. L. P. (2020c). Data from: Positive and negative interspecific interactions between coexisting rice planthoppers neutralise the effects of elevated temperatures Dryad Digital Repository, 10.5061/dryad.m37pvmd0h PMC7883635 33612910

[fec13683-bib-0018] Horgan, F. G. , Naik, B. S. , Iswanto, E. H. , Almazan, M. L. P. , Ramal, A. F. , & Bernal, C. C. (2016). Responses by the brown planthopper, *Nilaparvata lugens*, to conspecific density on resistant and susceptible rice varieties. Entomologia Experimentalis et Applicata, 158, 284–294.

[fec13683-bib-0019] Horgan, F. G. , Srinivasan, T. S. , Bentur, J. S. , Kumar, R. , Bhanu, K. V. , Sarao, P. S. , Chien, H. , Almazan, M. , Bernal, C. , Ramal, A. , Ferrater, J. , & Huang, S. (2017). Geographic and research center origins of rice resistance to Asian planthoppers and leafhoppers: Implications for rice breeding and gene deployment. Agronomy, 7, e62 10.3390/agronomy7040062 PMC7371011 32704393

[fec13683-bib-0020] Horgan, F. G. , Srinivasan, T. S. , Naik, B. S. , Ramal, A. F. , Bernal, C. C. , & Almazan, M. L. P. (2016). Effects of nitrogen on egg‐laying inhibition and ovicidal response in planthopper‐resistant rice varieties. Crop Protection, 89, 223–230. 10.1016/j.cropro.2016.07.033 27812236 PMC5026402

[fec13683-bib-0021] Hullé, M. , Cœur d'cier, A. , Bankhead‐Dronnet, S. , & Harrington, R. (2010). Aphids in the face of global changes. Comptes Rendus Biologies, 333, 497–503. 10.1016/j.crvi.2010.03.005 20541161

[fec13683-bib-0032] IPCC. (2014). Climate change 2014: Synthesis report In IPCC (Ed.), Contribution of Working Groups I, II and III to the Fifth Assessment Report of the Intergovernmental Panel on Climate Change (p. 151). IPCC Retrieved from https://ar5‐syr.ipcc.ch/ipcc/ipcc/resources/pdf/IPCC_SynthesisReport.pdf

[fec13683-bib-0022] Jeffs, C. T. , & Lewis, O. T. (2013). Effects of climate warming on host–parasitoid interactions. Ecological Entomology, 38, 209–218. 10.1111/een.12026

[fec13683-bib-0023] Kanno, H. , & Fujita, Y. (2003). Induced systemic resistance to rice blast fungus in rice plants infested by white‐backed planthopper. Entomologia Experimentalis et Applicata, 107, 155–158. 10.1046/j.1570-7458.2003.00045.x

[fec13683-bib-0024] Kanno, H. , Hasegawa, M. , & Kodama, O. (2012). Accumulation of salicylic acid, jasmonic acid and phytoalexins in rice, *Oryza sativa*, infested by the white‐backed planthopper, *Sogatella furcifera* (Hemiptera: Delphacidae). Applied Entomology and Zoology, 47, 27–34. 10.1007/s13355-011-0085-3

[fec13683-bib-0025] Kanno, H. , Satoh, M. , Kimura, T. , & Fujita, Y. (2005). Some aspects of induced resistance to rice blast fungus, *Magnaporthe grisea*, in rice plants infested by white‐backed planthopper, *Sogatella furcifera* . Applied Entomology and Zoology, 40, 91–97.

[fec13683-bib-0026] Lin, Z.‐H. , Wu, C.‐H. , & Ho, C.‐K. (2018). Warming neutralizes host‐specific competitive advantages between a native and invasive herbivore. Scientific Reports, 8, e11130. 10.1038/s41598-018-29517-0 PMC6057923 30042428

[fec13683-bib-0027] Liu, Z. , & Han, Z. (2006). Fitness costs of laboratory‐selected imidacloprid resistance in the brown planthopper, *Nilaparvata lugens* Stål. Pest Management Science, 62, 279–282. 10.1002/ps.1169 16475223

[fec13683-bib-0028] Lu, X. , Huo, Z. , Shen, S. , Huang, D. , Wang, L. , Xiao, J. , Yu, C. X. (2012). Effects of climate warming on the northern distribution boundary of brown planthopper (*Nilaparvata lugens* (Stål)) overwintering in China. Chinese Journal of Ecology, 31, 1977–1983.

[fec13683-bib-0029] Matsumura, M. , & Suzuki, Y. (2003). Direct and feeding‐induced interactions between two rice planthoppers, *Sogatella furcifera* and *Nilaparvata lugens*: Effects on dispersal capability and performance. Ecological Entomology, 28, 174–182. 10.1046/j.1365-2311.2003.00498.x

[fec13683-bib-0030] Meisner, M. H. , Harmon, J. P. , & Ives, A. R. (2014). Temperature effects on long‐term population dynamics in a parasitoid–host system. Ecological Monographs, 84, 457–476. 10.1890/13-1933.1

[fec13683-bib-0031] Ntiri, E. S. , Calatayud, P.‐A. , Van Den Berg, J. , Schulthess, F. , & Le Ru, B. P. (2016). Influence of temperature on intra‐and interspecific resource utilization within a community of lepidopteran maize stemborers. PLoS One, 11, e0148735 10.1371/journal.pone.0148735 26859748 PMC4747504

[fec13683-bib-0033] Pan, H. , Preisser, E. L. , Su, Q. , Jiao, X. , Xie, W. , Wang, S. , Wu, Q. & Zhang, Y. (2016). Natal host plants can alter herbivore competition. PLoS One, 11, e0169142 10.1371/journal.pone.0169142 28030636 PMC5193396

[fec13683-bib-0034] Park, C.‐G. , Kim, K.‐H. , Park, H.‐H. , & Lee, S.‐G. (2013). Temperature‐dependent development model of white backed planthopper (WBPH), *Sogatella furcifera* (Horváth) (Homoptera: Delphacidae). Korean Journal of Applied Entomology, 52, 133–140.

[fec13683-bib-0035] Piyaphongkul, J. , Pritchard, J. , & Bale, J. (2012). Can tropical insects stand the heat? A case study with the brown planthopper *Nilaparvata lugens* (Stål). PLoS One, 7, e0029409 10.1371/journal.pone.0029409 PMC3257224 22253720

[fec13683-bib-0036] Satoh, M. , Gomi, K. , Matsumura, M. , Takabayashi, J. , Sasaki, K. , Ohashi, Y. , & Kanno, H. (2009). Whitebacked planthopper–induced disease resistance in rice In K. L. Heong & B. Hardy (Eds.), Planthoppers: New threats to the sustainability of intensive rice production systems in Asia (pp. 327–339). International Rice Research Institute.

[fec13683-bib-0037] Schädler, M. , Brandl, R. , & Haase, J. (2007). Antagonistic interactions between plant competition and insect herbivory. Ecology, 88, 1490–1498. 10.1890/06-0647 17601141

[fec13683-bib-0038] Srinivasan, T. S. , Almazan, M. L. P. , Bernal, C. C. , Ramal, A. F. , Subbarayalu, M. K. , & Horgan, F. G. (2016). Interactions between nymphs of *Nilaparvata lugens* and *Sogatella furcifera* (Hemiptera: Delphacidae) on resistant and susceptible rice varieties. Applied Entomology and Zoology, 51, 81–90. 10.1007/s13355-015-0373-4

[fec13683-bib-0039] Sun, Y. C. , Chen, F. J. , & Ge, F. (2009). Elevated CO_2_ changes interspecific competition among three species of wheat aphids: *Sitobion avenae*, *Rhopalosiphum padi,* and *Schizaphis graminum* . Environmental Entomology, 38, 26–34. 19791595 10.1603/022.038.0105

[fec13683-bib-0040] Trnka, M. , Muška, F. , Semerádová, D. , Dubrovský, M. , Kocmánková, E. , & Žalud, Z. (2007). European corn borer life stage model: Regional estimates of pest development and spatial distribution under present and future climate. Ecological Modelling, 207, 61–84. 10.1016/j.ecolmodel.2007.04.014

[fec13683-bib-0041] Tu, Z. , Ling, B. , Xu, D. , Zhang, M. , & Zhou, G. (2013). Effects of southern rice black‐streaked dwarf virus on the development and fecundity of its vector, *Sogatella furcifera* . Virology Journal, 10, e145. 10.1186/1743-422X-10-145 PMC3698214 23663428

[fec13683-bib-0042] Wang, B. , Zhou, G. , Xin, Z. , Ji, R. , & Lou, Y. (2015). (Z)‐3‐Hexenal, one of the green leaf volatiles, increases susceptibility of rice to the white‐backed planthopper *Sogatella furcifera* . Plant Molecular Biology Reporter, 33, 377–387. 10.1007/s11105-014-0756-7

[fec13683-bib-0043] Wang, C. , Fei, M. , Meng, L. , Harvey, J. A. , & Li, B. (2020). Effects of elevated CO_2_ and temperature on survival and wing dimorphism of two species of rice planthoppers (Hemiptera: Delphacidae) under interaction. Pest Management Science, 76, 2087–2094. 31944534 10.1002/ps.5747

[fec13683-bib-0044] Wang, L. , Li, L.‐K. , Song, Y.‐Y. , Han, T. , Li, Z. , Wan, G.‐J. , & Chen, F. J. (2018). Elevated CO_2_ and temperature alter specific‐species population dynamics and interspecific competition of three wheat aphids. Journal of Applied Entomology, 142, 863–872.

[fec13683-bib-0045] Wang, Y. , Wang, X. , Yuan, H. , Chen, R. , Zhu, L. , He, R. , & He, G. (2008). Responses of two contrasting genotypes of rice to brown planthopper. Molecular Plant‐Microbe Interactions, 21, 122–132. 10.1094/MPMI-21-1-0122 18052889

[fec13683-bib-0046] Warton, D. I. , & Hui, F. K. C. (2011). Arcsine is asinine: The analysis of proportions in ecology. Ecology, 92, 3–10. 21560670 10.1890/10-0340.1

[fec13683-bib-0047] Wu, Y. , Gong, Z. , Bebber, D. P. , Miao, J. , Zhao, Z. , Jiang, Y. , Xiao, S. , Zhang, G. , Yu, D. , Fang, J. , & Lu, X. (2019). Phenological matching drives wheat pest range shift under climate change. bioRxiv, 614743 10.1101/614743

