# Supplementary material for: Positive and negative interspecific interactions between coexisting rice planthoppers neutralise the effects of elevated temperatures
Source: Funct Ecol. 2020 Oct 4;35(1):181–92. doi: 10.1111/1365-2435.13683 (PMC7883635; doi:10.1111/1365-2435.13683)
Supplement: Supplementary file 1 — Supplementary Material [file FEC-35-181-s001.pdf]

Functional Ecology

Two herbivores are better than one as temperatures increase in Asian rice fields

Finbarr G. Horgan, Arriza Arida, Goli Ardestani, Maria Liberty P. Almazan

Global warming can directly affect insects by expanding their distribution ranges poleward and by accelerating their development to produce more generations in a single season. Such positive effects on herbivorous insects are often predicted to increase damage to plants, including crops. However, negative interactions between species could dampen the direct positive effects of climate change on insect herbivores.

The brown planthopper, *Nilaparvata lugens*, is one of the principal pests of rice in Asia. The species has high survival, high fecundity and rapid development at temperatures of about 30°C. A second pest, the whitebacked planthopper, *Sogatella furcifera*, coexists with the brown planthopper on rice throughout Asia, but temperatures of about 30°C are detrimental to its survival, development and growth. Whitebacked planthoppers can more effectively colonize and feed on rice plants in the presence of brown planthoppers. In contrast, brown planthopper growth, development and fecundity are all supressed in the presence of whitebacked planthoppers.

We examined interactions between the two planthoppers at 25°C (favourable for whitebacked planthoppers) and 30°C (detrimental for whitebacked planthoppers but optimal for brown planthoppers). We expected that whitebacked planthoppers would have little impact on brown planthoppers at the higher temperature. However, contrary to expectations, brown planthoppers were more severely affected by whitebacked planthoppers at 30°C than at 25°C. This suggests that whitebacked planthoppers induce rice defences against brown planthoppers that are more effective at higher temperatures. As a consequence, both planthopper species had reduced growth, development, and fecundity where they occurred together at the higher temperature.

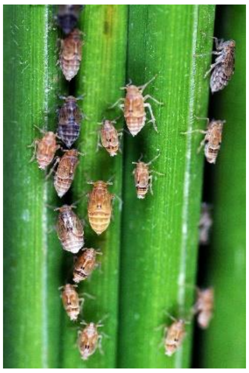

Brown planthopper nymphs feeding on rice. Photo courtesy of the International Rice Research Institute.

Simulations based on the life-histories of both species in single species and mixed populations indicated that rice plant mediated competition between whitebacked and brown planthoppers will supress total planthopper abundance on rice at both 25 and 30°C. This study presents one example of how the direct impacts of global warming on herbivore damage to crops may be avoided by maintaining biodiversity in agroecosystems.
