# Supplementary material for: Positive and negative interspecific interactions between coexisting rice planthoppers neutralise the effects of elevated temperatures
Source: Funct Ecol. 2020 Oct 4;35(1):181–92. doi: 10.1111/1365-2435.13683 (PMC7883635; doi:10.1111/1365-2435.13683)
Supplement: Supplementary file 2 — Supplementary Material [file FEC-35-181-s002.docx]

Supplementary information

**TABLE S1** Treatments and replicates in environmental chamber experiments

| Experiment | Temperature  (°C) | Life stage | Host plants | Number of BPH^1^ | Number of WBPH^2^ | Treatment cages per run^3^ | Run time (days) | Cages per experiment^4^ | Number of sub-replicates | Cages per chamber | Number of runs | Total treatment cages^5^ |
| --- | --- | --- | --- | --- | --- | --- | --- | --- | --- | --- | --- | --- |
| Temperature profile | 25 | Gravid female | IR22, T65 | 1 | 0 | 2 | 20 | 20 | 3 to 5 | 120 to 200 | 4 | 640 |
| Temperature profile | 30 | Gravid female | IR22, T65 | 1 | 0 | 2 | 20 | 20 | 3 to 5 | 120 to 200 | 4 | 640 |
| Temperature profile | 35 | Gravid female | IR22, T65 | 1 | 0 | 2 | 20 | 20 | 3 to 5 | 120 to 200 | 4 | 640 |
| Temperature profile | 25 | Gravid female | IR22, T65 | 0 | 1 | 2 | 20 | 20 | 3 to 5 | 120 to 200 | 4 | 640 |
| Temperature profile | 30 | Gravid female | IR22, T65 | 0 | 1 | 2 | 20 | 20 | 3 to 5 | 120 to 200 | 4 | 640 |
| Temperature profile | 35 | Gravid female | IR22, T65 | 0 | 1 | 2 | 20 | 20 | 3 to 5 | 120 to 200 | 4 | 640 |
| Temperature profile | 25 | Neonates | IR22, T65 | 10 | 0 | 2 | 15 | 1 | 3 | 6 | 5 | 30 |
| Temperature profile | 30 | Neonates | IR22, T65 | 10 | 0 | 2 | 15 | 1 | 3 | 6 | 5 | 30 |
| Temperature profile | 35 | Neonates | IR22, T65 | 10 | 0 | 2 | 15 | 1 | 3 | 6 | 5 | 30 |
| Temperature profile | 25 | Neonates | IR22, T65 | 0 | 10 | 2 | 15 | 1 | 3 | 6 | 5 | 30 |
| Temperature profile | 30 | Neonates | IR22, T65 | 0 | 10 | 2 | 15 | 1 | 3 | 6 | 5 | 30 |
| Temperature profile | 35 | Neonates | IR22, T65 | 0 | 10 | 2 | 15 | 1 | 3 | 6 | 5 | 30 |
| Competition | 25 | Gravid female | IR22, T65 | 0,2,12 | 4 | 6 | 7 | 1 | 2 | 56 | 5 | 280 |
| Competition | 25 | Gravid female | IR22, T65 | 2,12 | 0 | 4 | 7 | 1 | 4 |  |  |  |
| Competition | 25 | Gravid female | IR22, T65 | 4 | 0,2,12 | 6 | 7 | 1 | 2 |  |  |  |
| Competition | 25 | Gravid female | IR22, T65 | 0 | 2,12 | 4 | 7 | 1 | 4 |  |  |  |
| Competition | 30 | Gravid female | IR22, T65 | 0,2,12 | 6 | 6 | 7 | 1 | 2 | 56 | 5 | 280 |
| Competition | 30 | Gravid female | IR22, T65 | 2,12 | 0 | 4 | 7 | 1 | 4 |  |  |  |
| Competition | 30 | Gravid female | IR22, T65 | 6 | 0,2,12 | 6 | 7 | 1 | 2 |  |  |  |
| Competition | 30 | Gravid female | IR22, T65 | 0 | 2,12 | 4 | 7 | 1 | 4 |  |  |  |
| Competition | 25 | Neonates | IR22, T65 | 0,5,15 | 10 | 6 | 15 | 1 | 1 to 2 | 40 | 5 | 200 |
| Competition | 25 | Neonates | IR22, T65 | 5,15 | 0 | 4 | 15 | 1 | 1 to 2 |  |  |  |
| Competition | 25 | Neonates | IR22, T65 | 10 | 0,5,15 | 6 | 15 | 1 | 1 to 2 |  |  |  |
| Competition | 25 | Neonates | IR22, T65 | 0 | 5,15 | 4 | 15 | 1 | 1 to 2 |  |  |  |
| Competition | 30 | Neonates | IR22, T65 | 0,5,15 | 10 | 6 | 15 | 1 | 1 to 2 | 40 | 5 | 200 |
| Competition | 30 | Neonates | IR22, T65 | 5,15 | 0 | 4 | 15 | 1 | 1 to 2 |  |  |  |
| Competition | 30 | Neonates | IR22, T65 | 10 | 0,5,15 | 6 | 15 | 1 | 1 to 2 |  |  |  |
| Competition | 30 | Neonates | IR22, T65 | 0 | 5,15 | 4 | 15 | 1 | 1 to 2 |  |  |  |

1: Numbers are BPH densities per plant

2: Numbers are WBPH densities per plant

3: Host plant × density combinations

4: Plants with gravid females were destructively sampled each day after living females were transferred to new plants

5: A total of 4980 cages were sampled during the experiments

**TABLE S2** Results of general linear models for adult female longevity, oviposition and nymph development in environmental chambers at three constant temperatures (see Figures 1 and 2).

| Sources of variation | DF^1^ | F-values^2^ |  |  |  |  |  |
| --- | --- | --- | --- | --- | --- | --- | --- |
|  |  | Adult longevity | Eggs laid | Nymph survival | Development to fifth instar | Total planthopper weight | Average planthopper weight |
| Temperature (T) | 2 | 5.954** | 0.702 | 14.900*** | 3.014 | 133.293*** | 86.750*** |
| Species (S) | 1 | 2.843 | 37.112*** | 3.620 | 22.778*** | 21.959*** | 19.230*** |
| Variety (V) | 1 | 0.223 | 0.003 | 0.001 | 0.377 | 0.230 | 0.136 |
| Replicate | 3 [4] | 0.925 | 5.825*** | 1.795 | 5.700*** | 4.370*** | 4.700*** |
| Plant weight | 1 | 0.072 | 0.028 | 0.046 | 5.997* | 0.004 | 0.131 |
| T*S | 2 | 2.180 | 4.824* | 3.171* | 23.399*** | 28.843*** | 25.790*** |
| S*V | 1 | 3.225 | 0.353 | 0.057 | 0.295 | 0.003 | 0.097 |
| T*V | 2 | 1.664 | 1.718 | 1.025 | 0.984 | 2.107 | 1.259 |
| T*S*V | 2 | 0.521 | 2.504 | 1.344 | 0.064 | 0.311 | 0.025 |
| Error | 43 [32] |  |  |  |  |  |  |

1: DF in parentheses are for nymph survival experiments (i.e., nymph survival, development to fifth instar, total planthopper weight and average planthopper weight)

2: *** = P < 0.001, ** = P < 0.01, * = P < 0.05; data for survival were arcsine-transformed, data for development were ranked and weight data were log-transformed before analysis

**FIGURE S1** Numbers of eggs laid per plant (A-D) and per female (E-H) for BPH (A,B,E,F) and WBPH (C,D,G,H) at constant densities (4 per plant at 25°C,and 6 per plant at 30°C) in the absence (0) and presence (2 and 12 per plant) of heterospecifics. The survival of BPH (I,J) and WBPH (K,L) nymphs, the proportion of BPH (M,N) and WBPH (O,P) nymphs, and the total biomass of BPH (Q,R) and WBPH (S,T) nymphs at constant densities (10 per plant) in the absence (0) and presence (5 and 15 per plant) of heterospecifics are also indicated. Corresponding individual BPH (U,V) and WBPH (W,X) nymph biomass is also indicated. Data for BPH is presented as brown symbols and lines, data for WBPH as blue symbols and lines. Square symbols and solid lines indicate 25°C and circles with dashed lines are 30°C. Experiments were conducted on IR22 (A,C,E,G,I,K,M,O,Q,S,U,W) and T65 (B,D,F,H,J,L,N,P,R,T,V,X). Standard errors are indicated (N=5). Corresponding data for heterospecifics at increasing densities are presented in Figures 3, 4, S2, and S4.

**TABLE S3** Results from general linear models for effects of intra- and interspecific competition on oviposition by the brown planthopper (BPH) and whitebacked planthopper (WBPH) in single species and mixed infestations of IR22 and T65 rice plants. (See Figures 3, S1 and S2)

| Source of variation | DF | F-values^3^ |  |  |  |  |  |  |  |
| --- | --- | --- | --- | --- | --- | --- | --- | --- | --- |
| Planthopper species |  | BPH |  |  |  | WBPH |  |  |  |
| Parameter |  | Batches/plant | Eggs/plant | Batches/female | Eggs/female | Batches/plant | Eggs/plant | Batches/female | Eggs/female |
| *Test species (varying conspecific density)^1^* | | | |  |  |  |  |  |  |
| Variety (V) | 1 | 0.029 | 2.825 | 0.007 | 2.684 | 2.265 | 1.595 | 2.906 | 1.596 |
| Temperature (T) | 1 | 0.507 | 0.239 | 0.519 | 0.243 | 0.131 | 8.501*** | 0.297 | 8.368*** |
| Conspecific density (D) | 1 | 126.159*** | 139.607*** | 40.354*** | 39.858*** | 75.735*** | 75.98*** | 8.096** | 12.788*** |
| Heterospecific presence (Hp)^2^ | 1 | 3.401* | 4.517* | 3.337 | 4.539* | 5.537* | 0.467 | 5.127* | 0.452 |
| Replicate | 4 | 3.826* | 3.031* | 3.775** | 3.030* | 5.727*** | 9.392*** | 5.751*** | 10.729*** |
| Plant weight | 1 | 2.925 | 0.871 | 3.08 | 0.834 | 6.917** | 6.085* | 7.159** | 7.274** |
| V*D | 1 | 4.314* | 5.310* | 4.155* | 5.190* | 1.307 | 3.715 | 1.706 | 3.607 |
| T*D | 1 | 0.048 | 0.005 | 0.06 | 0.006 | 0.249 | 0.43 | 0.718 | 0.479 |
| D*Hp | 1 | 0.050 | 0.227 | 0.024 | 0.212 | 0.793 | 8.998*** | 0.442 | 8.876*** |
| V*T | 1 | 0.080 | 0.597 | 0.098 | 0.619 | 1.789 | 1.804 | 2.472 | 1.638 |
| V*Hp | 1 | 0.025 | 0.01 | 0.012 | 0.007 | 0.791 | 6.735* | 1.176 | 6.886** |
| T*Hp | 1 | 0.232 | 0.106 | 0.305 | 0.138 | 0.0001 | 1.95 | 0.03 | 2.216 |
| V*T*D | 1 | 0.198 | 0.227 | 0.218 | 0.235 | 0.351 | 6.879** | 0.311 | 7.221** |
| V*D*Hp | 1 | 0.385 | 0.522 | 0.34 | 0.518 | 1.195 | 1.979 | 1.602 | 1.976 |
| T*D*Hp | 1 | 2.253 | 3.365 | 2.163 | 3.308 | 1.764 | 3.501 | 1.123 | 2.836 |
| V*T*Hp | 1 | 0.042 | 0.827 | 0.025 | 0.805 | 3.845* | 1.027 | 4.197* | 0.739 |
| V*T*D*Hp | 1 | 0.361 | 0.289 | 0.342 | 0.27 | 0.01 | 7.237** | 0.053 | 8.024** |
| Error | 59 |  |  |  |  |  |  |  |  |
| *Competitor (constant density)^1^* |  | WBPH |  |  |  | BPH |  |  |  |
| Variety (V) | 1 | - | - | 2.707 | 0.033 | - | - | 0.289 | 2.595 |
| Temperature (T) | 1 | - | - | 10.329** | 8.536** | - | - | 1.054 | 0.134 |
| Heterospecific number (Hn)^2^ | 2 | - | - | 4.988* | 4.446* | - | - | 4.127* | 8.338*** |
| Replicate | 4 | - | - | 11.194*** | 5.242*** | - | - | 4.884*** | 5.067*** |
| Plant weight | 1 | - | - | 0.33 | 0.19 | - | - | 0.897 | 0.52 |
| V*Hn | 2 | - | - | 0.171 | 1.7 | - | - | 0.289 | 0.19 |
| T*Hn | 2 | - | - | 3.222* | 5.122** | - | - | 1.035 | 0.684 |
| V*T | 1 | - | - | 0.016 | 0.412 | - | - | 1.692 | 1.861 |
| V*T*Hn | 2 | - | - | 0.067 | 1.108 | - | - | 1.735 | 0.639 |
| Error | 43 |  |  |  |  |  |  |  |  |

1: Note that for each reciprocal experiment, analyses for test species batches or eggs and competitor batches or eggs are presented in the same table column; total batches or eggs per plant for the competitor at constant density are not presented because different numbers of females were used for experiments at 25°C and 30°C

2: Note that heterospecific presence (Hp) has two levels – present or absent, heterospecific number (Hn) has three levels (0, 2 and 12)

3: *** = P < 0.001, ** = P < 0.01, * = P < 0.05; data were log-transformed before analysis

**TABLE S4** Results from general linear models for effects of intra- and interspecific competition on nymph survival and development of the brown planthopper (BPH) and whitebacked planthopper (WBPH) in single species and mixed infestations on IR22 and T65 rice plants. (See Figure 4, S1 and S4)

| Source of variation | DF | F-values^3^ |  |  |  |  |  |  |  |
| --- | --- | --- | --- | --- | --- | --- | --- | --- | --- |
| Planthopper species |  | BPH |  |  |  | WBPH |  |  |  |
| Parameter |  | Nymph survival | Nymph development | Total nymph biomass | Individual nymph weight | Nymph survival | Nymph development | Total nymph biomass | Individual nymph weight |
| *Test species (varying conspecific density)^1^* | | | |  |  |  |  |  |  |
| Variety (V) | 1 | 0.718 | 0.693 | 1.532 | 1.317 | 1.932 | 0.009 | 0.568 | 2.081 |
| Temperature (T) | 1 | 2.868 | 3.043 | 10.935*** | 9.880*** | 22.033*** | 12.519*** | 271.314*** | 227.640*** |
| Conspecific density (D) | 1 | 1.081 | 3.471 | 617.701*** | 0.037 | 1.622 | 0.219 | 265.792*** | 0.130 |
| Heterospecific presence (Hp)^2^ | 1 | 1.303 | 2.390 | 4.839* | 5.318* | 4.117* | 2.624 | 11.751*** | 8.337*** |
| Replicate | 5 | 0.788 | 2.276 | 4.835*** | 4.403*** | 0.326 | 0.117 | 1.209 | 1.872 |
| Plant weight | 1 | 0.002 | 3.931* | 3.352 | 4.010* | 0.256 | 0.212 | 2.941 | 5.790* |
| V*D | 1 | 0.259 | 0.650 | 1.278 | 1.051 | 1.160 | 0.709 | 3.384 | 2.580 |
| T*D | 1 | 0.936 | 9.046*** | 3.589 | 6.097* | 0.005 | 0.001 | 14.217*** | 0.261 |
| D*Hp | 1 | 1.652 | 2.352 | 0.034 | 0.505 | 7.736** | 0.630 | 1.887 | 0.474 |
| V*T | 1 | 0.027 | 3.696 | 0.827 | 0.914 | 2.409 | 0.858 | 0.872 | 0.860 |
| V*Hp | 1 | 0.258 | 4.518* | 1.873 | 1.634 | 0.465 | 0.243 | 0.716 | 1.239 |
| T*Hp | 1 | 0.139 | 47.909*** | 10.602*** | 10.853*** | 2.641 | 4.718* | 1.916 | 1.391 |
| V*T*D | 1 | 1.058 | 3.049 | 0.108 | 0.035 | 0.822 | 1.173 | 0.675 | 0.276 |
| V*D*Hp | 1 | 1.369 | 0.447 | 0.280 | 0.151 | 1.139 | 0.118 | 0.351 | 0.717 |
| T*D*Hp | 1 | 1.534 | 10.378*** | 1.026 | 0.273 | 0.046 | 0.058 | 0.059 | 0.428 |
| V*T*Hp | 1 | 0.079 | 0.120 | 0.001 | 0.001 | 4.059* | 0.643 | 3.945* | 3.390 |
| V*T*D*Hp | 1 | 3.126 | 2.315 | 0.039 | 0.016 | 0.001 | 3.502 | 6.297** | 5.291* |
| Error | 74 |  |  |  |  |  |  |  |  |
| *Competitor (constant density)^1^* |  | WBPH |  |  |  | BPH |  |  |  |
| Variety (V) | 1 | 0.025 | 1.639 | 0.961 | 0.097 | 0.909 | 0.800 | 0.612 | 0.558 |
| Temperature (T) | 1 | 28.488*** | 20.373*** | 518.399*** | 605.483*** | 7.018** | 0.361 | 7.453** | 6.399** |
| Heterospecific number (Hn)^2^ | 2 | 0.148 | 2.611 | 7.875*** | 10.977*** | 1.490 | 0.364 | 2.736 | 2.813 |
| Replicate | 5 | 0.331 | 0.570 | 0.700 | 0.937 | 1.151 | 0.504 | 0.443 | 0.545 |
| Plant weight | 1 | 0.034 | 3.690 | 0.570 | 0.277 | 0.157 | 2.050 | 0.001 | 0.001 |
| V*Hn | 2 | 1.863 | 0.821 | 0.792 | 0.656 | 0.163 | 0.721 | 3.746* | 3.485* |
| T*Hn | 2 | 0.716 | 0.074 | 16.309*** | 19.995*** | 6.707*** | 2.365 | 5.774*** | 5.855*** |
| V*T | 1 | 0.011 | 0.008 | 2.641 | 2.243 | 0.270 | 0.027 | 0.006 | 0.792 |
| V*T*Hn | 2 | 1.190 | 1.135 | 4.912** | 5.458** | 0.899 | 1.839 | 0.674 | 0.545 |
| Error | 54 |  |  |  |  |  |  |  |  |

1: Note that for each reciprocal experiment, analyses for test species and competitors are presented in the same table column

2: Note that heterospecific presence (Hp) has two levels – present or absent, heterospecific number (Hn) has three levels (0, 5 and 15)

3: *** = P < 0.001, ** = P < 0.01, * = P < 0.05; survival and development data were arcsine-transformed and weights were log-transformed before analysis

**FIGURE S2** Results of oviposition from reciprocal interspecific competition experiments conducted at 25°C (A,C,E,G) and 30°C (B,D,F,H). In one set of experiments, WBPH female densities were held constant at 4 per plant (25°C) or 6 per plant (30°C) under increasing densities of BPH females (A-H), whereas in the second set, BPH female densities were held constant at 4 per plant (25°C) or 6 per plant (30°C) under increasing densities of WBPH females (E-H). Results indicate the total numbers of eggs for each planthopper species. The experiments were conducted using the varieties IR22 (A,B,E,F) and T65 (C,D,G,H). Brown symbols and lines indicate BPH and blue symbols and lines indicate WBPH. Standard errors are indicated. The numbers of eggs per female are presented in the main text (Figure 3). Results of LM analyses are indicated in Table S3.

**Index of competition effect**

To further assess the relative impacts of BPH or WBPH on heterospecific egg laying and nymph weight, we calculated the ‘index of competition effect’ as the reduction in the value of each parameter for individual planthoppers in the presence of heterospecifics, divided by the value of the parameter for an individual heterospecific (i.e., for oviposition: index of competition effect = [eggs produced per female in single-species infestation – eggs produced per female in the presence of heterospecifics]/eggs produced per heterospecific in two-species infestation). Indices were calculated for each species at high and low conspecific densities on each rice variety and at each temperature. Calculated indices were analysed using univariate GLM. Results are presented in Figures S3 and S5 and in Table S5.

**FIGURE S3** Changes in the average number of eggs produced per BPH (brown bars) or WBPH (blue bars) as a result of interspecific competition (i.e., index of competition effect). The effects of heterospecifics on each species are indicated for planthoppers on IR22 at 25°C (A) and 30°C (B), and on T65 at 25°C (C) and 30°C (D). Standard errors are indicated (N = 5).

**TABLE S5** Results of general linear models for indices of competition effect in the oviposition and nymph development experiments (see Figures S3 and S5)

| Sources of variation | DF | F-values^1^ |  |
| --- | --- | --- | --- |
|  |  | Egg number | Nymph weight |
| Temperature (T) | 1 | 0.867 | 5.344* |
| Variety (V) | 1 | 0.701 | 0.282 |
| Species (S) | 1 | 3.746* | 8.475*** |
| Conspecific density (D) | 1 | 0.034 | 1.97 |
| T*V | 1 | 0.308 | 0.038 |
| T*S | 1 | 1.058 | 4.992* |
| T*D | 1 | 0.568 | 1.284 |
| V*S | 1 | 1.326 | 0.008 |
| V*D | 1 | 0.297 | 0.145 |
| T*V*S | 1 | 1.568 | 0.341 |
| T*V*D | 1 | 0.629 | 1.113 |
| T*S*D | 1 | 0.841 | 1.785 |
| V*S*D | 1 | 0.931 | 0.778 |
| T*V*S*D | 1 | 0.124 | 0.014 |
| Error | 64 |  |  |

1: *** = P < 0.001, * = P < 0.05; data were log-transformed before analysis

**FIGURE S4** Total nymph biomass from reciprocal interspecific competition experiments conducted at 25°C (A,C,E,G) and 30°C (B,D,F,H) The experiments were conducted on varieties IR22 (A,B,E,F) and T65 (C,D,G,H). Brown symbols and lines indicate BPH and blue symbols and lines indicate WBPH. Standard errors are indicated (N = 5). The average weights of individual nymphs are presented in the main text (Figure 4Q-X). Results from LM analyses are presented in Table S4.

**FIGURE S5** Changes in the average weight of BPH (brown bars) or WBPH (blue bars) nymphs as a result of interspecific competition (i.e., index of competition effect). The effects of heterospecifics on each species are indicated for planthoppers on IR22 at 25°C (A) and 30°C (B), and on T65 at 25°C (C) and 30°C (D). Standard errors are indicated (N = 5).


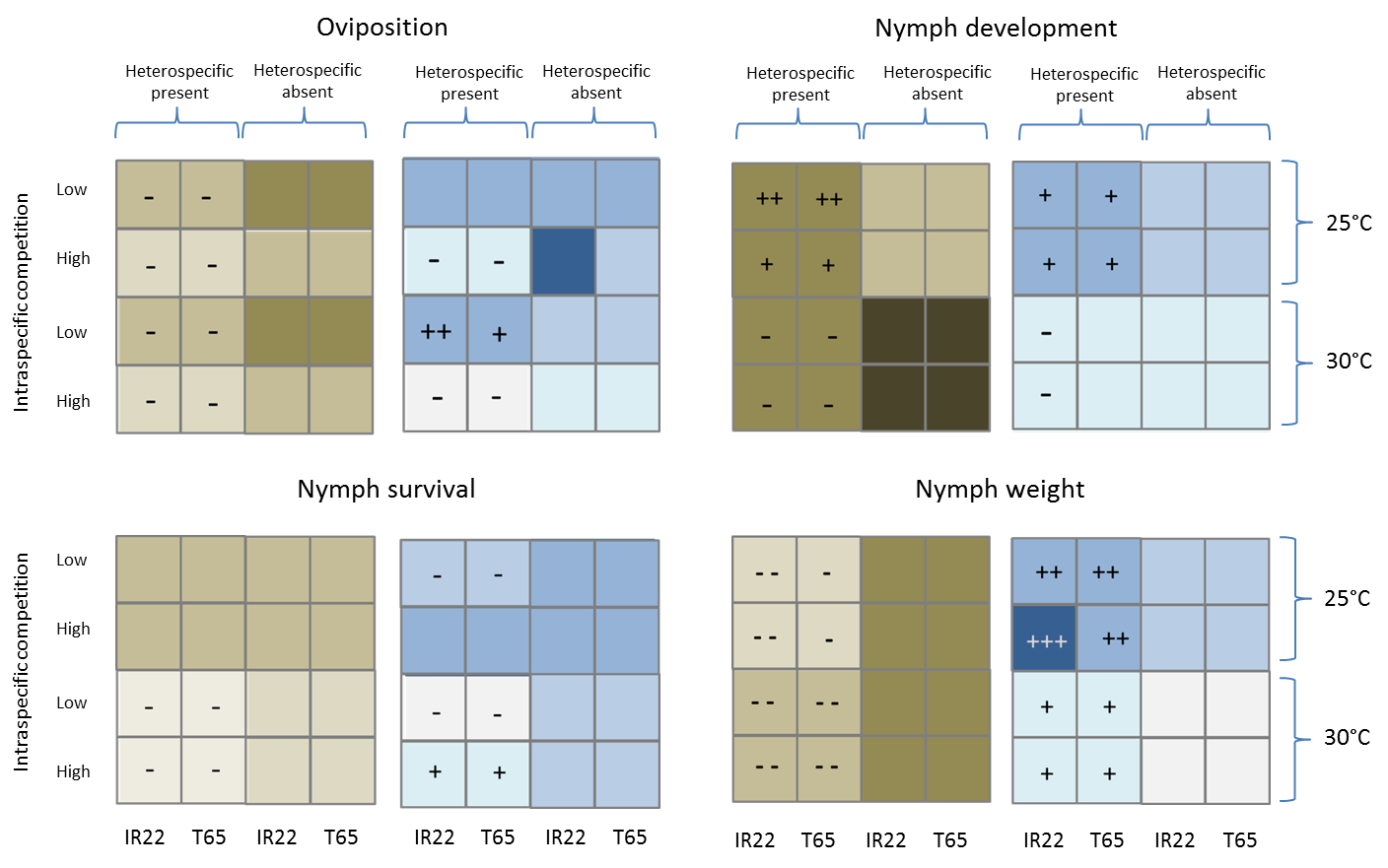


**FIGURE S6** Results from interspecific competition experiments summarized for BPH (brown squares) and WBPH (blue squares). Colour intensity indicates planthopper fitness from low (light colours) to high (dark colours). Pluses (+) and minuses (-) indicate effects of interspecifc crowding (stronger effects are represented by repeated symbols, i.e., ++ or +++). The figure demonstrates a general negative impact of WBPH on BPH egg-laying (indicated by minuses) and facilitation of WBPH by BPH (indicated by plus signs) at low conspecific densities at 30°C. The results as summarized from Figures 3, 4 and S1 indicate better performance of WBPH nymphs at 25°C and better development and weight gain of BPH nymphs at 30°C. The presence of heterospecifics increased WBPH survival at high conspecific densities, increased development at 25°C, and generally increased weight gain (at 25 and 30°C). For BPH nymphs, the presence of heterospecifics reduced survival and development at 30°C, but increased development at 25°C. Heterospecifics reduced BPH weight gain under all conditions.
